# Paroxysmal sympathetic hyperactivity and refractory hypotension in Guillain-Barré syndrome with autoimmune encephalitis: a case report and literature review

**DOI:** 10.3389/fnins.2025.1534771

**Published:** 2025-02-11

**Authors:** Jun-Ping Wang, Yan-Bo He

**Affiliations:** Department of Intensive Care Unit, Beijing Hui Min Hospital, Beijing, China

**Keywords:** Guillain-Barré syndrome, autoimmune encephalitis, autonomic dysfunction, paroxysmal sympathetic hyperactivity, refractory hypotension

## Abstract

Guillain-Barré syndrome (GBS) is an acute inflammatory peripheral nerve disorder mediated by autoimmune mechanisms. However, its co-occurrence with autoimmune encephalitis (AE) is rare. We present a 51-year-old man who initially presented with symmetrical numbness and weakness in all limbs, followed by hallucinations, behavioral abnormalities, and consciousness disturbances. Cerebrospinal fluid (CSF) analysis revealed protein-cell dissociation, indicative of GBS. Brain magnetic resonance imaging (MRI) showed abnormal signals in the splenium of the corpus callosum. Electromyography showed reduced amplitude in motor nerve conduction of bilateral common peroneal nerves and left tibial nerves. He developed respiratory distress, requiring tracheal intubation and mechanical ventilation. Finally, he was diagnosed with GBS combined with AE and received treatment with intravenous immunoglobulin (IVIG) and plasma exchange (PLEX). Subsequently, he experienced paroxysmal sympathetic hyperactivity (PSH) and refractory hypotension requiring vasopressor support. After comprehensive treatment, he was successfully weaned off the ventilator, and his refractory hypotension resolved after more than six months. This case illustrates that severe autonomic dysfunction can occur at any stage of GBS companied with AE. Furthermore, these patients often require prolonged ICU stays and experience slower recovery, but may still achieve a favorable outcome with appropriate integrated therapy.

## 1 Introduction

Autonomic dysfunction is common in Guillain–Barré syndrome (GBS), occurring in approximately two-thirds of cases. The manifestations of autonomic dysfunction are varied and may indicate hyper- or hypo-functioning of the sympathetic and/or parasympathetic nervous systems (Golden and Vernino, 2019). There have been no reports of Paroxysmal Sympathetic Hyperactivity (PSH) occurring in patients with GBS. Autoimmune encephalitis (AE) generally refers to a class of encephalitis mediated by autoimmune mechanisms, which often presents with autonomic dysfunction (Günther et al., 2016). PSH has a varying incidence of 9.1–50% after anti-N-methyl-d-aspartate receptor encephalitis (Wang et al., 2021; Chen et al., 2022). However, the simultaneous occurrence of GBS and AE is rare. Here, we present a case of a patient who experienced both GBS and AE, characterized by paroxysmal sympathetic hyperactivity and refractory hypotension.

## 2 Case report

**Figure 1:**
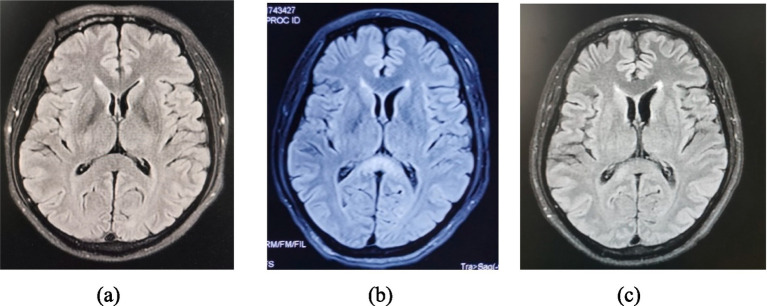
MRI brain FLAIR showed a hyperintense signal in the splenium on the 20 days **(A)**, 37 days **(B)** and 5.5 months **(C)** of illness, with obvious improvement of lesions splenium.

A 51-year-old male was admitted on May 19, 2023, with the chief complaints of “symmetrical numbness and weakness in all limbs for 19 days “. He had a history of a positive nucleic acid test for COVID-19 prior to the onset of symptoms. On May 1, 2023, the patient experienced numbness and coolness in the extremities, along with pain in the limbs and trunk. The following day, weakness in the lower limb muscle occurred; tendon reflexes except for bilateral biceps reflexes (+) were diminished; sensation in the limbs decreased in a glove-and-sock distribution, and bilateral pathological signs were negative. On May 7, the patient developed abdominal pain and ceased passing gas and stools. Abdominal computed tomography (CT) suggested an intestinal obstruction. On the 10th day, lumbar puncture revealed a normal cerebrospinal fluid (CSF) cell count but elevated CSF protein (95.6 mg/dL), supporting a diagnosis of Guillain-Barré syndrome. After receiving immunoglobulin therapy (IVIG, 0.4 g/kg /d for 5 days), the patient experienced relief from numbness, coolness, and pain. On May 12, the patient experienced intermittent sweating, extrapyramidal orofacial and hands dyskinesia during sleep and bilateral facial paralysis. On May 20, nocturnal hallucinations, intermittent agitation, shouting, and verbal abuse occurred, accompanied by disorientation in time, place, and person, consciousness impairment, and head magnetic resonance imaging (MRI) (Figure 1A) indicated abnormal signals in the splenium of the corpus callosum. Electromyography on the 23rd day showed reduced amplitude in motor conduction of the bilateral common peroneal nerves and left tibial nerves; the latency period of F-wave in the right tibial nerve prolonged. A repeat lumbar puncture on the 25th day showed protein-cell dissociation in CSF, and comprehensive antibody testing for neural gangliosides, central nervous system demyelinating antibodies, autoimmune encephalitis antibodies, and paraneoplastic syndrome antibodies in CSF and blood were all negative, which indicating a diagnosis of antibody-negative autoimmune encephalitis (AE). On May 27, the patient developed respiratory distress, requiring tracheal intubation and mechanical ventilation (MV), and was subsequently admitted to the neurological intensive care unit (NCU). The APACHE II score was 19. Mechanical ventilation was managed in volume-controlled mode with the following parameters: oxygen concentration 50%, tidal volume 500 mL, respiratory rate 18 breaths per minute, and positive end-expiratory pressure (PEEP) of 7 cmH₂O. Two cycles of plasma exchange (PLEX) and a second course of IVIG (0.4 g/kg /d, for 5 days) were administered, along with supportive medical care, including enteral nutrition support (25–30 kcal/kg/day), electrolyte supplementation, B-complex vitamins, and other supportive measures. The patient was diagnosed with GBS combined with AE. Two months after onset, Rituximab was administered once but was discontinued due to intolerance to side effects of severe pulmonary infection. After the above treatment, the patient’s consciousness returned to alertness, but he remained on MV in synchronized intermittent mandatory ventilation (SIMV) mode, with the following ventilator settings: oxygen concentration 45%, tidal volume 475 mL, respiratory rate 14 breaths per minute, PEEP of 5 cmH₂O, and pressure support (PS) of 12 cmH₂O and had cognitive impairment. Muscle strength graded as 3 in the distal upper limbs, 0 in both the proximal upper limbs and lower limbs.

On July 26, the patient developed intermittent sweating and facial flushing, bilateral pupil dilation, enhanced abdominal breathing, decreased pulse oxygen saturation and inaudible bilateral lung breath sounds. Additionally, there were no significant fluctuations in heart rate and blood pressure during each episode. Each episode lasted from a few minutes to one hour and occurred several times a day. There was no epileptic discharge on the electroencephalogram. Intravenous diazepam injection provided a relief, suggesting a possible diagnosis of paroxysmal sympathetic hyperactivity (PSH) (Baguley et al., 2014). According to the 2014 definition and diagnostic criteria for PSH, the patient’s PSH assessment measure (PSH-AM) was 4 points, and the diagnosis likelihood tool (DLT) was 11 points, with a total score of 15 points. Clonazepam 1 mg Q12H was added, resulting in a reduction in the frequency of the episode. In the later phase of the disease, when the above paroxysmal symptoms reoccurred, there was an increase in heart rate, respiratory rate, and blood pressure. The PSH-AM was 6 points, and the DLT was 11 points, resulting in a total score of 17 points, further supporting the diagnosis of PSH.

**Figure 2:**
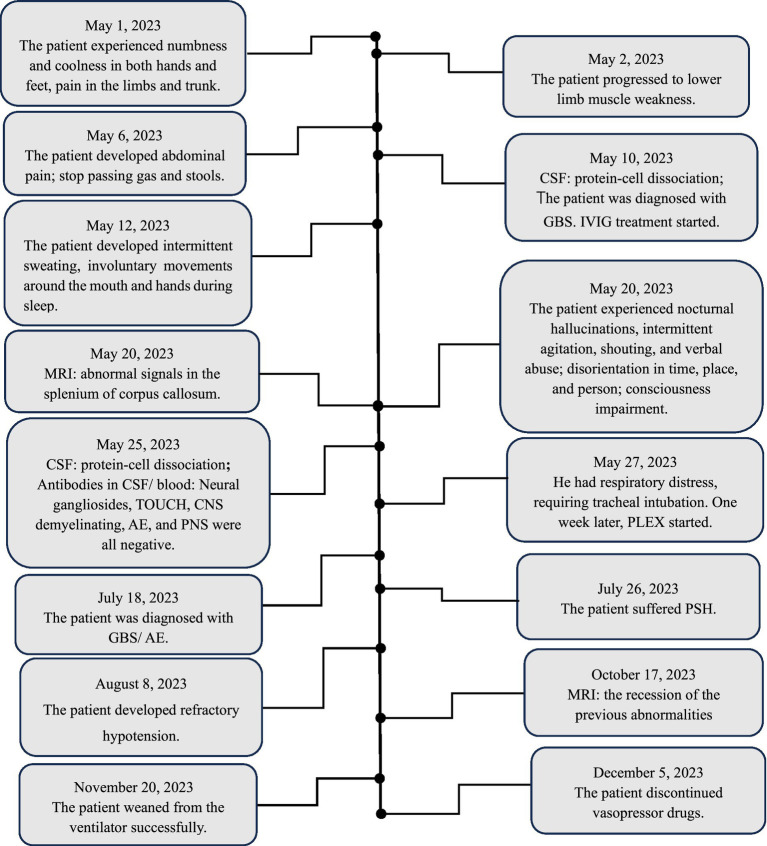
Patient timeline. CSF: cerebrospinal fluid; WBC: white blood cell; CNS: central nervous system; PNS: paraneoplastic syndrome; GBS: Guillain-Barré syndrome; IVIG: intravenous immunoglobulin; MRI: magnetic resonance imaging; AE: autoimmune encephalitis; PLEX: plasma exchange; PSH: paroxysmal sympathetic hyperactivity.

Around on August 8, the patient developed refractory hypotension necessitating vasopressor support, with Sequential Organ Failure Assessment (SOFA) score 4 (with vasopressor doses >0.1 μg/kg/min). On September 15, abdominal distension, intermittent gastric residue and gas were observed, and abdominal CT suggested an incomplete intestinal obstruction. Treatment included continuous administration of vasopressor drugs, monthly IVIG administration (0.4 g/kg /d for 5 days, for a total of 3 cycles), nutritional support, gastrointestinal decompression, and MV. Intermittent off-ventilator training began in the fifth month of onset (October 17), and ventilator weaning was successfully achieved in the sixth month after onset (November 20). Refractory hypotension was resolved, and vasoactive medications were discontinued in the seventh month of onset (December 5). Repeated MRI (Figures 1B,C) revealed the regression of the previous abnormalities. Muscle strength in the upper limbs improved to grade 3 in the proximal and to grade 4 in the distal; in the lower limbs, it improved to grade 2. Figure 2 shows the course of the disease.

## 3 Discussion

The co-occurrence of GBS and AE is rare. The pathogenesis of this combined condition may be related to COVID-19 infection. Stoian et al. have shown that COVID-19 is associated with the development of autoimmune disorders, including GBS and AE (Stoian et al., 2021; Stoian et al., 2022). However, autonomic nervous system abnormalities are commonly reported as pathophysiological features in both GBS and AE. Bazán-Rodríguez et al. (2023) showed that 31% of GBS patients experienced autonomic dysfunction, with manifestations including hypertension (84.8%), hypotension (76.1%), tachycardia (76.1%), gastrointestinal dysmotility (76.1%), and the need for vasopressor drugs (58.7%). One study suggested that GBS patients with severe autonomic dysfunction tended to have longer hospital stays, a higher likelihood of ICU admission, and severer disability at discharge (Chakraborty et al., 2020). The incidence of autonomic dysfunction in AE is not clear. He and Lian (2023) showed that 104 out of 164 AE patients (64.6%) presented autonomic dysfunction. Sinus tachycardia was the most common autonomic dysfunction, followed by pollakiuria/uroclepsia, central hypoventilation, sinus bradycardia, constipation, uroschesis, hyperhidrosis, hypersalivation, hypotension, which increase morbidity and mortality, complicate intensive care and could lead to hemodynamic shock (Byun et al., 2015). Our patient encountered GBS and AE in succession, which could promote drastic autonomic disorders.

Throughout the illness, our patient progressed from atypical to typical episodes of PSH. We supposed that this phenomenon resulted from severe injury to the peripheral sympathetic nervous system during the early stages of GBS, followed by gradual recovery of the peripheral sympathetic system and prominent excitatory symptoms of the central sympathetic nervous system due to AE. Interestingly, the patient was initially responsive to diazepam, but this effect diminished over time, likely related to long-term use of clonazepam, leading to drug tolerance. This phenomenon has also been reported in previous studies (Chen et al., 2022). As is well known, PSH is most commonly seen in traumatic brain injury but can also occur in ischemic–hypoxic encephalopathy, cerebrovascular diseases, anti-NMDA receptor encephalitis, and stroke (Muraoka et al., 2023). Research by Hinson et al. noted an association between PSH and lesions in the corpus callosum and posterior limb of the internal capsule (Hinson et al., 2015). The patient had a lesion in the corpus callosum, which gradually shrank with immunotherapy, and PSH episodes disappeared, supporting the notion that PSH was caused by AE. However, the patient exhibited an overlap of autonomic dysfunction typically associated with GBS and PSH induced by AE. This dual pathology was responsible for the atypical presentation of PSH symptoms and highlights the uniqueness of this case, as it illustrates the complex interplay between these two conditions.

Blood pressure variability in GBS patients can manifest as hypertension, transient hypotension, or refractory hypotension (Zochodne, 1994). Dysregulation of the sympathetic and parasympathetic nervous systems could cause changes in vascular tone and peripheral vascular resistance, leading to blood pressure fluctuations (Zochodne, 1994; Pfeiffer, 1999). Drastic fluctuations between hypotension and hypertension in GBS patients could result in cardiovascular collapse (Zaeem et al., 2019). In our patient, refractory hypotension occurred approximately 3 months from onset, requiring vasopressor support and accompanied by significant fluctuations in heart rate, which is relatively rare in GBS. The coexistence of AE may be a contributing factor. Hanxin et al. (2020) described a case of a patient with refractory hypotension who suffered from GABA(B) receptor autoimmune encephalitis. Ha et al. (2017) reported that severe bradycardia and refractory hypotension were the initial manifestations in a GBS patient. Despite receiving a temporary pacemaker, the patient eventually died of multiple organ failure due to the refractory hypotension. Fortunately, our patient responded well to vasopressor therapy and was able to discontinue vasopressor drugs as the condition improved.

Immunotherapy is a crucial treatment for both GBS and AE. For GBS patients with evident autonomic nervous system dysfunction, it remains unclear whether PLEX or IVIG can prevent or alleviate the severity of autonomous nervous abnormalities (Chakraborty et al., 2020). In this patient, after two rounds of PLEX and multiple sessions of IVIG treatment, there has been a partial recovery in neurological function, especially in respiratory muscle strength, allowing the patient to be weaned off MV, discontinued vasoactive medications, and experience a disappearance of PSH episodes. A follow-up cranial MRI revealed a significant reduction in abnormal signals in the corpus callosum, and the patient was able to stand up with the assistance of walking aids three months after discharge. This case demonstrates that these patients often require longer hospitalizations in the intensive care unit and experience a slower recovery. However, they have a high chance of achieving a favorable outcome with appropriate integrated treatment.

## Data Availability

The raw data supporting the conclusions of this article will be made available by the authors, without undue reservation.
